# Efficacy of Empirical Radioiodine Therapy in Patients with Differentiated Thyroid Cancer and Elevated Serum Thyroglobulin without Evidence of Structural Disease: A Propensity Score Analysis

**DOI:** 10.3390/cancers15164196

**Published:** 2023-08-21

**Authors:** Leandra Piscopo, Emilia Zampella, Fabio Volpe, Valeria Gaudieri, Carmela Nappi, Paolo Cutillo, Federica Volpicelli, Maria Falzarano, Leonardo Pace, Alberto Cuocolo, Michele Klain

**Affiliations:** 1 Department of Advanced Biomedical Sciences, University of Naples, Federico II, 80131 Naples, Italy; leandra.piscopo@unina.it (L.P.); emilia.zampella@unina.it (E.Z.); fabio.volpe@unina.it (F.V.); valeria.gaudieri@unina.it (V.G.); c.nappi@unina.it (C.N.); paolo.cutillo@unina.it (P.C.); fed.volpicelli@studenti.unina.it (F.V.); maria.fal@hotmail.it (M.F.); cuocolo@unina.it (A.C.); 2 Department of Medicine Surgery and Dentistry, University of Salerno, 84081 Salerno, Italy; lpace@unisa.it

**Keywords:** differentiated thyroid cancer, thyroglobulin, radioactive iodine therapy, prognosis, empiric therapy, propensity score

## Abstract

**Simple Summary:**

In patients with differentiated thyroid cancer, the standard treatment consists in surgery followed by radioactive iodine (RAI) therapy. Follow-up is usually performed by serum thyroglobulin measurements and neck ultrasound, in order to detect the presence of persistent disease. Detectable thyroglobulin levels after the first treatment may indicate the presence of still viable tumor cells. Therefore, empiric radioiodine administration can be considered for both diagnostic and therapeutic purposes in the presence of elevated thyroglobulin after the first treatment also in patients without evidence of persistent disease. A beneficial effect of this approach has been suggested, in particular in patients with high suspicious of distant metastases at post-therapy whole body scan. However, a significant impact on patient outcomes has not been addressed andthe identification of patients who may benefit from this approach has not been fully clarified. Therefore, the use of empiric RAI therapy in patients with DTC and its potential impact on outcome still remains controversial.

**Abstract:**

We assessed the outcome of administration of empiric radioactive iodine (RAI) therapy to patients with differentiated thyroid cancer (DTC), in a propensity-score-matched cohort of patients with biochemical incomplete response (BIR) and without evidence of structural disease. We retrospectively evaluated 820 DTC patients without distant metastases, who underwent total thyroidectomy followed by RAI therapy, with available BIR at 12 months and follow-up evaluations. The patients were categorized according to the administration of empiric therapy (ET). To account for differences between patients with (*n* = 119) and without (*n* = 701) ET, a propensity-score-matched cohort of 119 ET and 119 no-ET patients was created. The need for additional therapy and the occurrence of structural disease were considered as end-points. During a median follow-up of 53 months (range 3–285), 57 events occurred (24% cumulative event rate). The rate of events was significantly higher in the no-ET compared to the ET patients (30% vs. 18% *p* < 0.001). The multivariate Cox analysis identified age (*p* < 0.01), pre-therapy Tg (*p* < 0.05) and empiric RAI therapy (*p* < 0.01) as predictors of outcome. The Kaplan–Meier analysis found that progression-free survival was lower in no-ET patients compared to the ET group (*p* < 0.01). In patients with DTC treated with surgery and RAI, and with biochemical incomplete response at the 12-month evaluation, their prognosis seemed to be affected by Tg values and the empiric treatment. The identification of candidates for this approach may improve prognosis.

## 1. Introduction

The standard treatment of patients with differentiated thyroid cancer (DTC) consists of surgery followed by radioactive iodine therapy (RAI) [1,2]. The response to initial therapy is usually assessed by serum thyroglobulin (Tg) levels and neck ultrasonography (US) according to American Thyroid Association (ATA) guidelines [3]. Despite abnormal Tg levels usually correlating with the presence of viable tumor cells, 15% of patients with detectable serum Tg levels will not show evidence of structural disease during follow-up [4,5]. Diagnostic iodine-131 (^131^I) whole body scan (WBS) has been largely used for assessment of disease status, but it has high specificity and low sensitivity and is less frequently performed [6,7]. Empiric RAI administration can be considered for both diagnostic and therapeutic purposes in patients with detectable Tg values after the first treatment and without evidence of structural disease. Despite this approach still being applied in clinical practice, a real benefit in terms of outcome has not been clearly proven and it remains controversial. A beneficial effect of ^131^I therapy in patients with DTC and elevated Tg values has been observed in patients with distant metastases on post-therapy WBS scans [8]. More recently, the improved prognosis of patients with detectable serum Tg levels but negative diagnostic WBS has been proven in patients who underwent empiric RAI treatment compared to those who did not [9]. The aim of this study was to assess the prognostic impact of empiric RAI therapy in a propensity-score-matched cohort of DTC patients, treated with surgery and RAI administration and who had detectable Tg serum levels during follow-up.

## 2. Materials and Methods

### 2.1. Study Population

**Figure 1:**
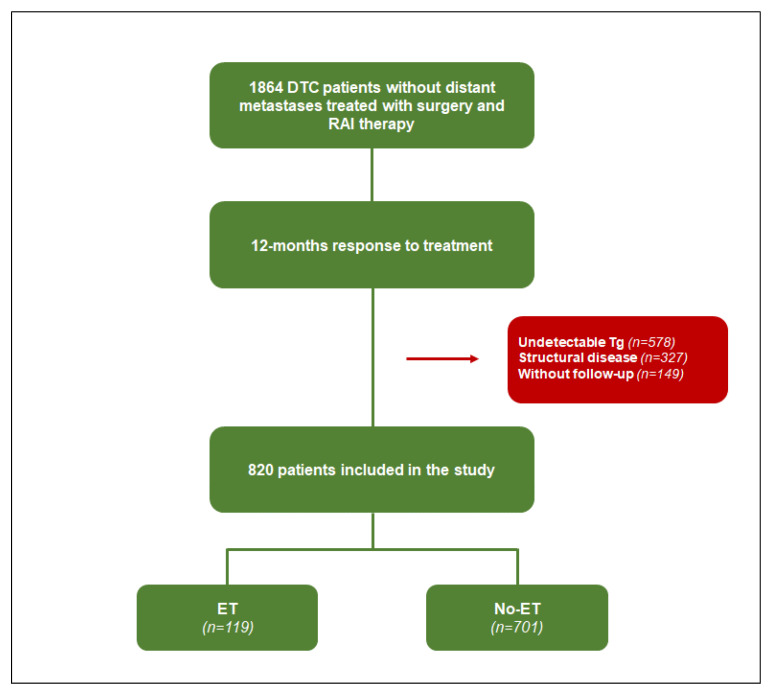
The study protocol. We considered 1864 DTC patients, without distant metastases, treated with surgery and RAI therapy. Of those, 820 patients with biochemical incomplete response at the 12-month evaluation were enrolled. A total of 119 patients underwent empiric RAI therapy and 701 did not.

A total of 1864 DTC patients, treated with a total thyroidectomy and RAI therapy in our institution (University Federico II, Naples, Italy) between 1992 and 2009 without evidence of distant metastases at initial evaluation were considered for this retrospective study. Clinical and histopathological data were collected and, according to the ATA guidelines, the patients were classified as low, intermediate, or high risk of structural recurrence [1,10]. The L-thyroxine withdrawal was performed before the RAI treatment, in order to increase the serum TSH level above a threshold of >30 mIU/l. Serum Tg was measured by a chemiluminescence system (Immulite, Diagnostic Products Corp., Los Angeles, CA, USA) with a detection limit of 0.2 ng/mL. RAI therapy was administered and five to seven days later, a post-therapy WBS was performed using a dual-head gamma camera (E.CAM, Siemens Medical Systems, Hoffman Estates, IL, USA) equipped with thick crystals and high-energy collimators. The response was evaluated at 12 months according to the 2015 ATA guidelines [3]. For the purposes of the present investigation, 820 patients with a biochemical incomplete response, defined as having a Tg ≥ 1 ng/mL on LT4 treatment or stimulated Tg ≥ 10 ng/mL, without evidence of structural disease and available subsequent follow-up were considered for the present study. Among these, a total of 119 patients were referred by clinical physician to empiric ^131^I therapy (ET) and 701 were not (no-ET). The study protocol is shown in Figure 1.

### 2.2. Follow-Up

After the 12-month evaluation, all patients were followed with serum Tg determinations (on L-thyroxine and in some patients off L-thyroxine therapy) and with imaging procedures every 6–12 months. Disease status was recorded at each evaluation. The progression-free survival (PFS) was considered as an end-point and measured from the date of empiric RAI administration in ET patients, or at the 12-month evaluation in the no-ET cohort, up to the date of first observation of progressive disease, relapse, need for additional therapy (i.e., ^131^I therapy or surgery) or death. Progression was defined as locoregional recurrence or metastases diagnosed by histology or an imaging procedure, new evidence of highly suspicious disease or imaging showing an increase in a known suspicious lesions. Patients last known to be alive and progression free were censored at the date of last contact.

### 2.3. Statistical Analysis

Continuous data are expressed as mean ± standard deviation and categorical data as percentage. Student’s two-sample *t* test and χ^2^ test were used to compare the differences in continuous and categorical variables, respectively. To create a matched cohort of patients with and without ET, a propensity score (logit model) was calculated for each individual based on their baseline clinical variables (age, sex, histology, ATA risk category and pre-therapy Tg values). The nearest available Mahalanobis metric matching method with caliper size specification (0.25 × standard deviation of propensity score) was used to perform one-to-one matched analysis without replacement using the Stata module PSMATCH2 [11]. Hazard ratios with 95% confidence intervals (CIs) were also calculated by univariate and multivariate Cox regression analyses. Variables showing a *p* value < 0.05 in the univariate analysis were considered for multivariate analysis. Survival analysis was performed using the Kaplan–Meier method and log-rank test. Statistical analyses were performed with Stata 12 software (StataCorp, College Station, TX, USA).

## 3. Results

### 3.1. Patients Characteristics before and after Matching

The baseline characteristics of the entire population according to empiric RAI therapy before and after propensity score matching are shown in Table 1. Before matching, pre-therapy Tg values and administered ^131^I activity, as well as the prevalence of high ATA risk and nodal involvement were higher in patients who underwent empiric RAI therapy compared to those who did not. Similarly, Tg values at the 12-month evaluation were higher in ET patients compared to those of the no-ET group. After matching, the clinical findings were comparable among the 119 patients who underwent empiric RAI treatment compared to the 119 subjects who did not.

### 3.2. Predictors of Outcome

During a median follow-up of 53 months (range 3–285 months), 57 patients experienced an event (24% cumulative event rate). Of these, 10 patients required additional RAI treatment for persistent disease in the thyroid bed, 15 for persistent disease in both the thyroid bed and lymph nodes, 9 for lung metastases and 3 for bone metastases. The remaining 22 patients with events underwent both additional surgery and RAI therapy for thyroid bed (*n* = 2) and nodal disease (*n* = 20). The clinical findings of the patients with and without events are reported in Table 2.

**Figure 2:**
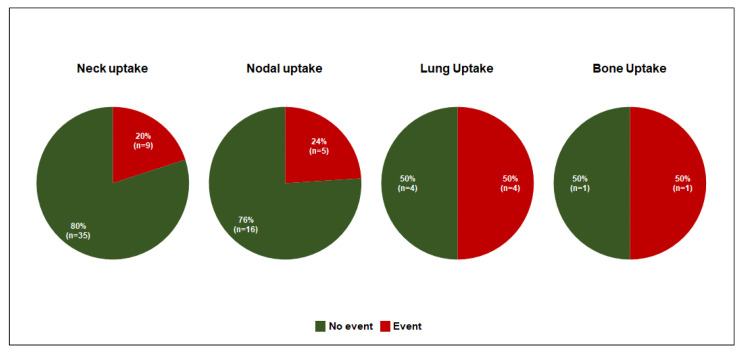
Outcomes of 75 patients who underwent empiric RAI treatment according to post-therapy WBS findings. In each graph, the percentage of patients with (red) and without (green) subsequent events after ET was reported according to post-therapy WBS results.

A total of 36 no-ET and 21 ET patients had events during subsequent follow-up. The rate of events was significantly higher in no-ET patients compared to those who underwent empiric RAI (30% vs. 18% *p* < 0.001). The outcomes of ET patients according to the post-therapy WBS scan acquired after empiric RAI are reported in Figure 2.

Age (*p* < 0.01), pre-therapy Tg values (*p* < 0.05) and empiric RAI therapy (*p* < 0.001) were identified as predictors of events by both the univariate and multivariate COX analyses (Table 3).

**Figure 3:**
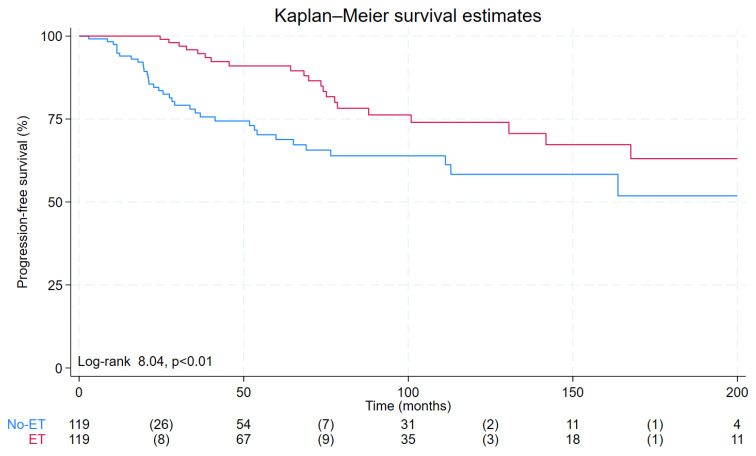
Progression-free survival (PFS) curves by Kaplan–Meier analysis according to empiric RAI treatment. No-ET patients (blue line) showed a significantly lower PFS compared to ET patients (red line) (175 ± 15 vs. 213 ± 14 months, *p* < 0.01).

The Kaplan–Meier analysis found that the progression-free survival was lower in no-ET patients compared to the ET group (175 ± 15 vs. 213 ± 14 months, *p* < 0.01) (Figure 3).

## 4. Discussion

In this retrospective study, we evaluated the efficacy of empiric RAI treatment in patients with DTC. In particular, in patients with biochemical incomplete response after 12 months and without evidence of structural disease after the first treatment, we found a significant improvement in outcome in those who underwent ET compared to no-ET patients.

In patients with DTC, the ATA dynamic risk stratification system has been introduced to monitor the effect of initial treatment and improve initial risk stratification [3,12]. Disease status can be monitored by serum Tg determination and imaging procedures 12 months after surgery and RAI therapy [3,13,14]. In patients without detectable Tg levels after the first treatment, prognosis is excellent and further diagnostic and therapeutic procedures can be avoided [7,15]. In patients with a biochemical incomplete response, the persistence of detectable Tg levels may reflect the presence of viable tumor cells. Therefore, further diagnostic procedures, such as a diagnostic WBS scan, may be considered in these patients. A significant proportion of patients with abnormal Tg levels may have a negative diagnostic WBS scan, without evidence of pathological uptake [7,16,17]. The management of these patients still remains controversial. Serum Tg may remain detectable after the initial treatment and subsequently disappear without any further interventions [18]. However, a higher rate of structural recurrence has been previously observed in these patients [19]. The administration of empiric RAI treatment can be considered in patients with abnormal serum Tg levels after the first treatment, for both diagnostic and therapeutic intents [1]. However, a real benefit of this approach in terms of outcome has not been fully demonstrated. Sabra et al. [5] evaluated 27 DTC patients with proven metastases and negative diagnostic WBS scan, who underwent empiric RAI treatment. The authors found that a significant proportion of patients showed stable cross-sectional imaging after RAI remnant ablation. In the 56% of the entire population, RAI administration was not able to treat structural disease progression. Therefore, they concluded that ET was not associated with regression of stable lesions, or conversion from progressive to stable disease. Similarly, Tramontin et al. [20] confirmed no significant differences during a long-term follow-up between treated and untreated patients. Likewise, Yuan et al. [9] evaluated the outcome of 80 DTC patients with elevated Tg levels after surgery and RAI treatment and negative diagnostic WBS. Among these 80 patients, 52 underwent ET and 28 did not; the authors found that ET was associated with an improved outcome.

In our report, we selected a cohort of 820 DTC patients, treated with surgery and RAI, that showed a biochemical incomplete response at 12 months after the first treatment. Of those, 119 were referred for ET and 701 were not. Several significant differences in baseline characteristics between the two population emerged from our data. It should be considered that the higher prevalence of patients with more aggressive disease may have affected the results.

Therefore, differently from previous reports [9], in order to account for differences between the two populations and reduce potential bias related to different risk profiles, a propensity score analysis was applied. Therefore, a final cohort of119 ET and 119 no-ET patients with balanced clinical variables was obtained. After subsequent treatment, the rate of subsequent structural events was higher in no-ET patients compared to ET. Our data are in agreement with previous studies [8,9,21] where the potential benefit of empiric treatment in terms of outcome were observed in particular in patients with distant metastases on post-therapy WBS scan. In our population, the patients with distant metastases at initial diagnosis have been excluded. However, the post-therapy WBS scan performed after empiric RAI showed neck uptake in 44 patients, nodal uptake in 21 patients and lung and bone metastases in 8 and 2 subjects, respectively. Of note, among the eight patients with lung metastases, only four underwent further RAI treatment during the subsequent follow-up. It should be considered that PET/CT imaging emerged has a strong complementary tool for the evaluation of patients with suspected DTC recurrence [22,23,24,25]. In our population, PET/CT has not been routinely performed in all patients; it should be considered that additional imaging findings would have had an impact on the selection of candidate patients for RAI treatment.

Different from a previous report [9], pre-therapy Tg values and age were identified as independent predictors of outcome in addition to empiric RAI treatment. Both age and pre-therapy Tg values have been previously recognized as strong predictors of outcome in patients with DTC [10,26]. Of note, in our cohort, the Tg values at the time of the 12-month evaluation did not show a significant association with outcome. In the survival analysis, ET patients showed a better prognosis compared to no-ET subjects.

The present study has some limitations related to its retrospective observational nature. The surveillance of DTC patients in our center is routinely performed according to ATA guidelines [1]. Therefore, other biochemical markers of disease were not available.

## 5. Conclusions

The empiric administration of RAI therapy has a beneficial effect on the outcomes of DTC patients. The ET patients have a lower risk of structural recurrence compared to no-ET patients. However, further data are needed in order to identify patients who may routinely benefit from this approach.

## Figures and Tables

**Table 1:** Baseline characteristics according to empiric RAI therapy before and after propensity score matching.

	Before Matching				After Matching			
	All Patients (*n* = 820)	No-ET (*n* = 701)	ET (*n* = 119)	*p* Value	All Patients (*n* = 238)	No-ET (*n* = 119)	ET (*n* = 119)	*p* Value
Age (years)	44 ± 15	44 ± 14	44 ± 17	0.69	45 ± 17	45 ± 17	44 ± 17	0.76
Female gender, *n* (%)	660 (80)	567 (81)	93 (78)	0.49	183 (77)	90 (76)	93 (78)	0.64
ATA risk categories								
Low risk, *n* (%)	201 (24)	181 (26)	20 (17)	<0.05	38 (16)	19 (16)	20 (17)	0.85
Intermediate risk, *n* (%)	438 (54)	382 (54)	56 (47)	0.11	105 (44)	48 (40)	56 (47)	0.29
High risk, *n* (%)	181 (22)	138 (20)	43 (36)	<0.001	95 (40)	52 (44)	43 (36)	0.23
Follicular type, *n* (%)	117 (14)	94 (13)	23 (19)	0.09	40 (17)	17 (14)	23 (19)	0.29
Tumor size >2 cm, *n* (%)	359 (44)	294 (42)	65 (55)	<0.01	140 (59)	75 (63)	65 (55)	0.19
Neck dissection, *n* (%)	241 (29)	197 (28)	44 (37)	<0.05	88 (37)	44 (37)	44 (37)	1
Lymph node involvement, *n* (%)	161 (20)	125 (18)	36 (30)	<0.01	68 (29)	32 (27)	36 (30)	0.57
Time interval surgery/RAI therapy (days)	132 ± 194	670 ± 61	124 ± 159	0.06	735 ± 480	125 ± 116	124 ± 159	0.37
Administered ^131^I activity (MBq)	3368 ± 1034	982 ± 38	1266 ± 119	<0.001	3652 ± 1073	3640 ± 871	1266 ± 119	0.769
Pre-therapy Tg (ng/mL)	34 ± 98	25 ± 83	88 ± 146	<0.001	74 ± 132	60 ± 115	88 ± 146	0.481
Neck uptake at WBS, *n* (%)	802 (98)	685 (98)	117 (98)	0.68	233 (98)	116 (97)	117 (98)	0.65
12-month follow-up Tg (ng/mL)	17 ± 19	17 ± 18	14 ± 18	0.67	12 ± 18	9 ± 19	14 ± 18	0.07

Data are presented as mean ± SD or number and percentage (%). Continuous and categorical variables were compared by student’s two-sample *t* test and χ^2^ test, respectively. ET, empiric therapy; Tg, thyroglobulin; WBS, post-therapy whole-body scan.

**Table 2:** Baseline characteristics according to the occurrence of events.

	Event (*n* = 57)	No Event (*n* = 181)	*p* Value
Age (years)	53 ± 17	42 ± 16	<0.001
Female gender, *n* (%)	38 (67)	145 (80)	<0.05
ATA risk categories			
Low risk, *n* (%)	6 (11)	33 (188)	0.17
Intermediate risk, *n* (%)	19 (33)	84 (46)	0.08
High risk, *n* (%)	32 (56)	64 (35)	<0.01
Follicular type, *n* (%)	25 (44)	88 (49)	0.52
Tumor size >2 cm, *n* (%)	35 (61)	105 (58)	0.65
Neck dissection, *n* (%)	26 (46)	62 (34)	0.12
Lymph node involvement, *n* (%)	21 (37)	47 (26)	0.11
Time interval surgery/RAI therapy (days)	140 ± 134	155 ± 550	0.74
Administered ^131^I activity (MBq)	3863 ± 1157	3580 ± 1037	0.08
Pre-therapy Tg (ng/mL)	109 ± 148	62 ± 125	<0.01
Neck uptake at WBS, *n* (%)	56 (98)	177 (98)	0.44
12-month follow-up Tg (ng/mL)	10 ± 17	15 ± 21	0.06
Empiric therapy, *n* (%)	21 (37)	98 (54)	<0.05
No empiric therapy, *n* (%)	36 (63)	83 (46)	<0.05

Data are presented as mean ± SD or number and percentage (%). Continuous and categorical data were compared by student’s two-sample *t* test and χ^2^ test, respectively. *Tg,* thyroglobulin; *WBS*, post-therapy whole-body scan.

**Table 3:** Univariate and multivariate predictors of events.

	Univariate		Multivariate	
	Hazard Ratio (95% CI)	*p* Value	Hazard Ratio (95% CI)	*p* Value
Age	10.3 (1.01–1.05)	<0.001	1.03 (1.01–1.05)	<0.01
Female sex	0.49 (0.28–0.86)	<0.05	0.62 (0.35–1.11)	0.11
ATA risk categories				
Low risk (reference)				
Intermediate risk	1.25 (0.61–3.83)	0.36	1.60 (0.62–4.12)	0.33
High risk	3.01 (1.22–7.24)	<0.05	2.04 (0.37–4.95)	0.12
Pre-therapy Tg ng/mL	1.00 (1.00–1.01)	<0.01	1.00 (1.00–1.01)	<0.05
Empiric RAI therapy	14.2 (4.11–48.7)	<0.001	0.43 (0.24–0.75)	<0.01

Tg, thyroglobulin obtained following thyroid hormone withdrawal before RAI administration.

## Data Availability

The data presented in this study are available on request from the corresponding author. The data are not publicly available due to privacy reasons.
